# Temporal Change: Evaluation of Brain Atrophy in Patients with Radiologically Isolated Syndrome Using volBrain

**DOI:** 10.3390/medicina62101948

**Published:** 2026-10-09

**Authors:** Furkan Akkoç, Aybala Neslihan Alagöz, Sena Destan Bünül, Burcu Alparslan, Hüsnü Efendi

**Affiliations:** 1 Department of Neurology, Faculty of Medicine, Kocaeli University, Kocaeli 41380, Türkiye; 2 Department of Radiology, Faculty of Medicine, Kocaeli University, Kocaeli 41380, Türkiye

**Keywords:** radiologically isolated syndrome, multiple sclerosis, brain atrophy, brain volume, longitudinal MRI, volBrain, neurodegeneration

## Abstract

***Background and Objective:*** Radiologically isolated syndrome (RIS) is characterized by magnetic resonance imaging (MRI) findings suggestive of multiple sclerosis (MS) in individuals without a history of clinical demyelinating events. Increasing evidence indicates that structural brain abnormalities may be present in RIS before the development of overt neurological symptoms. However, most previous studies have employed cross-sectional designs, limiting the ability to characterize the temporal evolution of neurodegenerative changes. This study aimed to investigate longitudinal changes in global, regional, and lobar brain volumes in individuals with RIS using serial MRI examinations obtained at least six months apart. ***Methods:*** Patients with RIS who had undergone two 3 Tesla (3T) brain MRI examinations separated by an interval of at least six months were retrospectively identified. Three-dimensional (3D) T1-weighted MRI scans obtained at both time points were analyzed using the volBrain pipeline of the volBrain platform to quantify global, regional, and lobar brain volumes. Longitudinal volumetric changes were expressed as annualized percentage volume changes. The number of T2 lesions and total T2 lesion volume were additionally assessed, and their associations with longitudinal brain volume changes were investigated. ***Results:*** A total of 28 patients with RIS were included. After correction for multiple comparisons using the Benjamini–Hochberg false discovery rate (FDR) procedure, significant annual volume loss was observed in total brain volume (*p* = 0.043), total cerebrum volume (*p* = 0.033), right cerebrum volume (*p* = 0.033), left cerebrum volume (*p* = 0.044), total thalamic volume (*p* = 0.033), right thalamic volume (*p* = 0.047), and left thalamic volume (*p* = 0.043). No significant associations were observed between annualized brain volume changes and follow-up duration, total or regional T2 lesion counts, or total T2 lesion volume. Similarly, annualized brain volume changes did not differ significantly between subgroups defined according to MRI progression, the presence of gadolinium-enhancing lesions, or the presence of spinal cord lesions. ***Conclusions:*** Longitudinal volumetric analysis demonstrated progressive brain volume loss involving both global and regional structures in patients with RIS. These findings provide further evidence of measurable longitudinal structural brain changes in RIS before the development of clinically overt demyelinating disease. Larger longitudinal studies incorporating appropriate control groups are warranted to further characterize the temporal trajectory of volumetric changes in RIS.

## 1. Introduction

Radiologically isolated syndrome (RIS) is defined by the incidental identification of white matter lesions characteristic of multiple sclerosis (MS) on magnetic resonance imaging (MRI) in individuals without a history of clinical demyelinating events or an alternative pathological explanation for the imaging findings. These lesions are most often detected during MRI examinations performed for unrelated neurological symptoms or incidentally in otherwise asymptomatic individuals [1]. Although RIS is clinically asymptomatic by definition, it is increasingly recognized as an early phase of the MS disease spectrum, as a subset of individuals subsequently develop a first clinical demyelinating event and meet diagnostic criteria for MS [2,3,4].

The rapid advancement of neuroimaging techniques, particularly the widespread availability of high-field MRI, has facilitated the recognition of RIS and enabled the detection of characteristic MS-like lesions before the onset of clinical manifestations. Consequently, RIS cohorts have provided a unique opportunity to investigate the earliest structural and pathological changes associated with MS. In recent years, an increasing number of studies have evaluated volumetric and tissue-specific alterations in individuals with RIS, with a smaller number employing longitudinal designs. These studies offer an opportunity to characterize structural brain changes during the clinically silent phase of the disease.

Quantitative assessment of brain structure has demonstrated that neurodegenerative changes can occur in MS alongside focal inflammatory and demyelinating lesions. Importantly, accumulating evidence suggests that such structural abnormalities may already be detectable during the RIS stage, before the emergence of overt clinical disease. Volumetric MRI studies have reported reductions in the volumes of specific brain structures in individuals with RIS, suggesting that neurodegenerative processes may begin during this clinically silent phase [5,6,7]. However, the available evidence remains limited, in part because RIS cohorts are generally small.

Moreover, most volumetric MRI studies in RIS have used cross-sectional designs. Although cross-sectional analyses can identify differences in brain volume between individuals or groups, they provide limited information regarding the rate and temporal evolution of tissue loss within individual patients. This distinction is particularly relevant when investigating early neurodegenerative processes, as detecting a difference in volume at a single time point does not establish whether or how rapidly structural changes are progressing. Longitudinal assessment of brain volume may therefore provide a more sensitive approach for characterizing the dynamics of neurodegeneration during the clinically silent phase of MS.

In the present study, we performed a longitudinal analysis of global, lobar, and regional brain volumes in individuals with RIS using serial MRI examinations obtained at least six months apart. Brain volumetry was performed using the volBrain platform, which enables automated segmentation and quantitative assessment of multiple brain structures. We aimed to determine whether measurable brain volume loss occurs over time in patients with RIS and to explore whether longitudinal volumetric changes are associated with MRI measures of disease burden, including T2 lesion counts, total T2 lesion volume, MRI progression, the presence of gadolinium-enhancing lesions, and spinal cord involvement. By characterizing the temporal and regional distribution of brain volume changes in RIS, we sought to provide further insight into the structural abnormalities that emerge before the development of clinically overt demyelinating disease.

## 2. Materials and Methods

### 2.1. Study Design and Participants

This retrospective cohort study included patients diagnosed with RIS according to the 2023 RIS criteria who were followed at the Neurology Outpatient Clinic of Kocaeli University.

Patients were eligible for inclusion if they had two brain MRI examinations performed at least six months apart on a 3T MRI scanner (Achieva; Philips Medical Systems, Best, The Netherlands) and if both examinations met the technical requirements for volumetric analysis using the volBrain platform.

Patients were excluded if they did not have two technically adequate 3T brain MRI examinations, if the available images were unsuitable for volBrain analysis, if they had a diagnosis of a demyelinating disease other than RIS, if neurological examination revealed objective findings attributable to a demyelinating disorder, or if they had a history of a clinical demyelinating attack.

Brain MRI follow-up examinations of patients followed at the Multiple Sclerosis Clinic of Kocaeli University are routinely performed using a standardized demyelinating disease MRI protocol. The protocol includes a three-dimensional (3D) T1-weighted sequence, which is acquired at both time points and used for longitudinal volumetric analysis.

### 2.2. MRI Data Acquisition and Preprocessing

3D T1-weighted and 3D fluid-attenuated inversion recovery (FLAIR) brain MRI sequences acquired at both time points were retrieved from the hospital imaging archive in Digital Imaging and Communications in Medicine (DICOM) format.

DICOM images were converted to Neuroimaging Informatics Technology Initiative (NIfTI) format using the dcm2niix conversion tool implemented in MRIcron (www.nitrc.org (accessed on 10 September 2026); developed by Chris Rorden, McCausland Center for Brain Imaging, University of South Carolina, Columbia, SC, USA).

The 3D T1-weighted images were subsequently processed using the volBrain pipeline of the volBrain platform to obtain automated measurements of global, regional, and lobar brain volumes. T2 lesion counts and total T2 lesion volume were obtained automatically from the 3D FLAIR images using the DeepLesionBrain module.

All segmentation outputs generated by volBrain were visually inspected for quality assurance by experienced investigators. Scans demonstrating substantial segmentation errors that could potentially affect volumetric measurements were excluded from the analysis. Images without significant segmentation abnormalities were retained, and all volumetric and lesion-related measurements generated by the analysis pipelines were included in the statistical evaluation.

### 2.3. Longitudinal Volumetric Assessment

For each participant, volumetric measurements obtained at the two MRI time points were compared to quantify longitudinal changes in brain volume. Changes in brain volume were expressed as annualized percentage volume changes, taking into account the interval between the two MRI examinations. Global, lobar, and regional volumetric measures were evaluated to characterize the spatial distribution of longitudinal brain volume loss.

The associations between longitudinal volumetric changes and radiological measures of disease burden were subsequently examined. These included the total number of T2 lesions, regional T2 lesion counts, total T2 lesion volume, MRI progression, the presence of gadolinium-enhancing lesions, and the presence of spinal cord lesions.

### 2.4. Volumetric and Lesion Assessment

**Figure 1:**
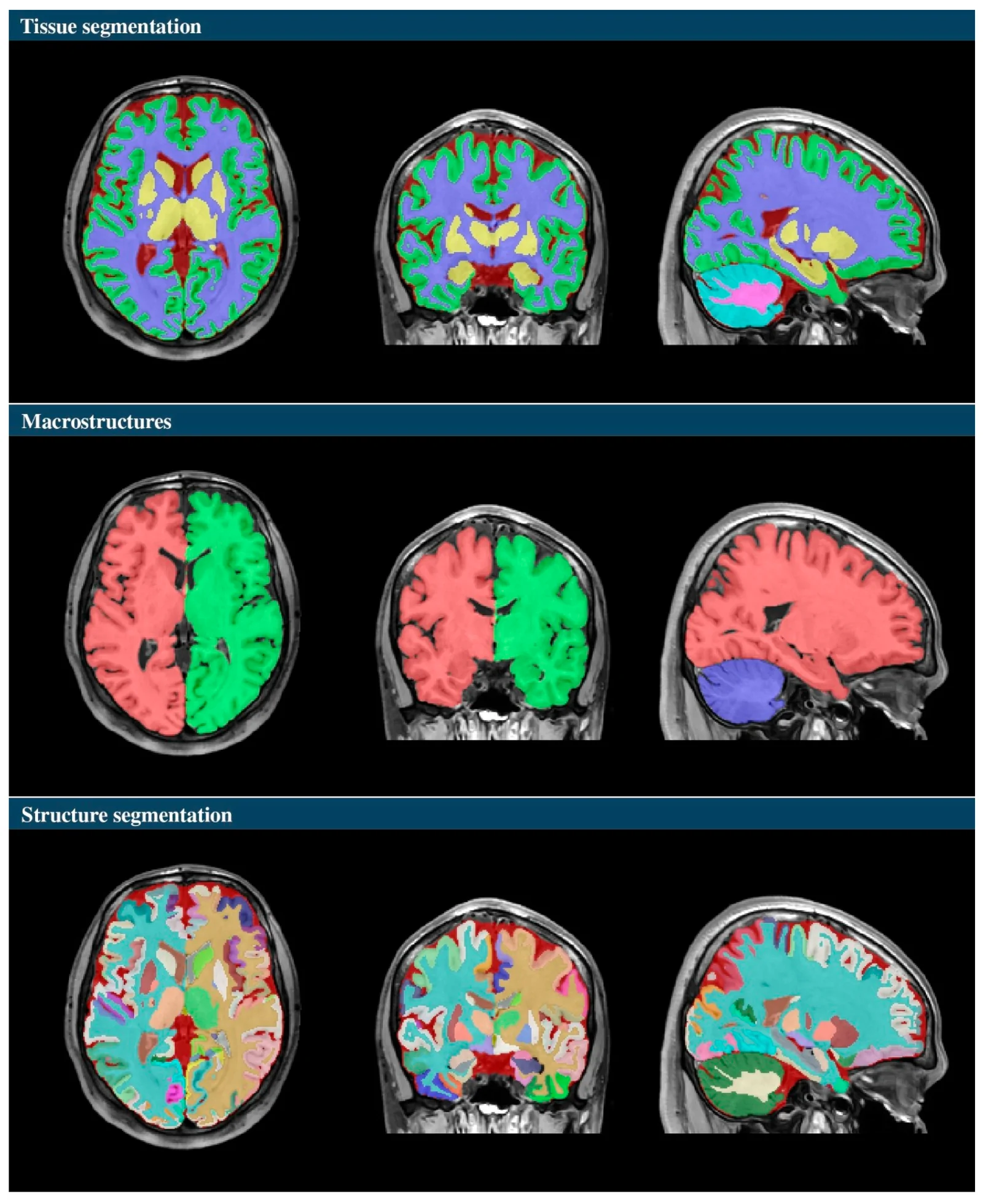
Post-processing images and representative examples of automated segmentation obtained using the volBrain-based analysis pipeline.

The analysis included global brain volume measures, including total white matter, total gray matter, total subcortical gray matter, total cortical gray matter, cerebrospinal fluid (CSF), total brain, total cerebrum, total cerebral white matter, and total cerebral gray matter volumes. Right and left hemispheric volumes were also assessed, including right cerebrum, right cerebral white matter, right cerebral gray matter, left cerebrum, left cerebral white matter, and left cerebral gray matter. Cerebellar volumes included total cerebellum, total cerebellar white matter, total cerebellar gray matter, right cerebellum, right cerebellar white matter, right cerebellar gray matter, left cerebellum, left cerebellar white matter, and left cerebellar gray matter. Subcortical structures, including the brainstem, accumbens, amygdala, basal forebrain, caudate, hippocampus, pallidum, putamen, thalamus, and ventral diencephalon, were also evaluated. In addition, cortical lobe volumes, including the frontal, temporal, parietal, occipital, limbic, and insular cortices, were assessed in detail.Representative images of the automated segmentation results obtained using the volBrain-based analysis pipeline are shown in Figure 1.

For each brain region, the change in volume was initially calculated as a percentage change from baseline. To account for differences in the duration of follow-up, all volumetric changes were subsequently converted to annualized percentage volume changes. The annualized percentage volume change was calculated using the following formula:

$$\mathrm{Annualized}\;\mathrm{Percentage}\;\mathrm{Volume}\;\mathrm{Change}\;(\%)\;=\frac{((t_{1}-t_{0})/t_{0})\times 100}{MR\;interval\;(months)/12}$$

where *t*_0_ represents the volume at the first time point and *t*_1_ represents the volume at the second time point.

The resulting annualized percentage volume change values were used for statistical analyses.

Lesion assessment was performed on 3D FLAIR images using the DeepLesionBrain module. For both time points, the total number of T2 lesions, as well as the number of periventricular, juxtacortical, and infratentorial T2 lesions and the total T2 lesion volume, were obtained automatically. The presence of gadolinium-enhancing lesions, MRI progression, and spinal cord lesions was also assessed at both *t*_0_ and *t*_1_. These parameters were recorded to characterize the imaging features of the cohort and were included in the statistical analyses.

For patients who successfully completed the volumetric analyses, clinical records were retrospectively reviewed through the hospital information system. Demographic characteristics, neurological examination findings, and disease-modifying therapy (DMT) use were recorded. Patients who fulfilled all inclusion and exclusion criteria constituted the final study cohort.

The study was approved by the Non-Interventional Clinical Research Ethics Committee of Kocaeli University Faculty of Medicine (Decision No. KÜ GOKAEK-2025/14/08; Project No. 2025/325; approved on 18 June 2025).

### 2.5. Statistical Analysis

Statistical analyses were performed using IBM SPSS Statistics version 29.0 (IBM Corp., Armonk, NY, USA) and R software 4.5.3 (R Foundation for Statistical Computing, Vienna, Austria). The normality of continuous variables was assessed using the Shapiro–Wilk test. As the assumption of normality was not met, continuous variables were presented as median values with interquartile ranges (IQRs; 25th–75th percentiles). Categorical variables were presented as frequencies and percentages [n (%)].

The Mann–Whitney U test was used for between-group comparisons, the Wilcoxon signed-rank test was used for paired comparisons, and Spearman rank correlation analysis was used to assess associations between variables. To account for differences in follow-up duration, volumetric changes were expressed as annualized percentage volume changes.

The Benjamini–Hochberg false discovery rate (FDR) correction was applied to control the risk of Type I error arising from multiple comparisons. Both unadjusted *p* values and FDR-adjusted *p* values were reported. Statistical significance was determined based on the FDR-adjusted *p* values, with an FDR-adjusted *p* value < 0.05 considered statistically significant.

## 3. Results

A total of 28 patients with RIS who were followed at the Multiple Sclerosis (MS) Outpatient Clinic of Kocaeli University and met the study eligibility criteria were included in the analysis. The demographic and clinical characteristics of the participants are presented in Table 1.

Of the patients included in the study, 89.3% (*n* = 25) were female and 10.7% (*n* = 3) were male. At the time of the MRI examination performed at *t*_0_, the median age was 41.00 years (IQR, 33.25–45.75). The median interval between the two MRI examinations was 15.50 months (IQR, 9.50–24.75). None of the patients was receiving DMT at the initial assessment.

Clinical records showed that SDMT scores were available for 22 patients, while results of the Timed 25-Foot Walk test and the Nine-Hole Peg Test for both the right and left hands were available for 24 patients. The median SDMT score was 43.50 (IQR, 30.25–51.25). The median Timed 25-Foot Walk test duration was 11.43 s (IQR, 8.78–13.65). The median Nine-Hole Peg Test duration was 20.81 s (IQR, 17.94–23.75) for the right hand and 20.65 s (IQR, 18.74–21.27) for the left hand.

The *t*_0_ and *t*_1_ volumetric measurements, annualized percentage volume changes, unadjusted *p* values, and FDR-adjusted *p* values for global and regional brain volumes showing significant longitudinal changes after FDR correction are presented in Table 2. The complete results for all assessed brain regions, including unadjusted and FDR-adjusted *p* values, are presented in Supplementary Table S1.

Following the Wilcoxon signed-rank test and correction for multiple comparisons using the FDR procedure, statistically significant decreases in annualized percentage volume change were observed for total brain volume (FDR-adjusted *p* = 0.043), total cerebrum volume (FDR-adjusted *p* = 0.033), right cerebrum volume (FDR-adjusted *p* = 0.033), left cerebrum volume (FDR-adjusted *p* = 0.044), total thalamic volume (FDR-adjusted *p* = 0.033), right thalamic volume (FDR-adjusted *p* = 0.047), and left thalamic volume (FDR-adjusted *p* = 0.043).

Among the regions demonstrating significant longitudinal volume changes, the greatest annualized volume loss was observed in the left thalamus (−2.6%), followed by the right thalamus (−2.2%), total thalamus (−1.9%), right cerebrum (−1.3%), total brain (−1.1%), total cerebrum (−1.1%), and left cerebrum (−1.1%).

The associations between annualized percentage volume changes in the brain regions demonstrating significant longitudinal changes and age and the interval between *t*_0_ and *t*_1_ were assessed using Spearman rank correlation analysis. The results are presented in Table 3.

Spearman rank correlation analysis was performed to assess the associations between annualized percentage volume changes and age and the interval between *t*_0_ and *t*_1_. No statistically significant associations were observed between the *t*_0_–*t*_1_ interval and annualized percentage volume changes. Similarly, no statistically significant correlations were identified between age and annualized percentage volume changes after FDR correction (Table 3).

The MRI lesion characteristics of the patients included in the study are presented in Table 4. Total and regional T2 lesion counts, total T2 lesion volume, MRI progression, and the presence of contrast-enhancing and spinal cord lesions were evaluated.

The associations between MRI lesion parameters and annualized brain volume changes were assessed using Spearman rank correlation analysis. No statistically significant correlations were observed between total lesion count, regional T2 lesion counts, or total T2 lesion volume and annualized brain volume changes after FDR correction (FDR-adjusted *p* > 0.05). The results are presented in Table 5.

Additional subgroup analyses were performed according to MRI progression, the presence of contrast-enhancing lesions during follow-up, and the presence of spinal cord lesions. The Mann–Whitney U test was used for between-group comparisons, whereas the Wilcoxon signed-rank test was used for within-group (*t*_0_–*t*_1_) comparisons. Although significant longitudinal volume changes were observed in several brain regions within some subgroups, no statistically significant differences in annualized brain volume changes were identified between the respective subgroups (Supplementary Tables S2–S5).

## 4. Discussion

In recent years, numerous volumetric studies have been published evaluating changes in brain volume in both multiple sclerosis (MS) and radiologically isolated syndrome (RIS). However, most of these studies have employed cross-sectional designs and have been limited to patient–control comparisons. In contrast, prospective studies in the MS literature have successfully demonstrated dynamic patterns of gray and white matter atrophy using repeated MRI examinations at two time points following diagnosis [8]. In RIS, however, longitudinal studies evaluating individual-level changes in brain volumes over time remain scarce. In the present study, each participant’s baseline MRI was used as their own reference, and changes in brain volume between two time points were assessed using volBrain segmentation. Differences in follow-up duration were standardized by calculating annualized percentage volume change. In addition, the FDR correction was applied for multiple comparisons to improve the reliability of the findings. This approach may contribute to a more robust assessment of regional volumetric changes over time in patients with RIS.

In line with these objectives, our study demonstrated significant annualized volume loss in the total brain, total cerebrum, right cerebrum, left cerebrum, total thalamus, right thalamus, and left thalamus volumes.

To minimize the potential effect of differences in follow-up duration, all volumetric changes in our study were calculated as annualized percentage volume change. This approach enabled a more standardized comparison among patients with different follow-up durations and allowed our findings to be compared with annual rates of atrophy reported in the literature. Furthermore, no significant correlation was observed between follow-up duration and annualized volume changes. Nevertheless, given the limited sample size, this finding cannot exclude a potential effect of follow-up duration on volumetric changes.

In the MS literature, annual brain volume loss in patients with relapsing-remitting multiple sclerosis (RRMS) has been reported to range from 0.6% to 1.35%, while untreated patients may experience up to 2.7% loss of brain parenchymal volume over a two-year period [9]. In contrast, annual brain volume loss associated with normal aging in healthy individuals is considerably lower [10,11]. The annualized losses in total brain and total cerebrum volumes observed in our study suggest that measurable changes in global brain volume may already occur during the RIS stage, before the development of a clinical demyelinating attack.

Although annualized percentage volume changes were standardized according to follow-up duration, direct comparisons across studies should be interpreted with caution because of differences in imaging protocols, segmentation methods, analytical software, and patient characteristics. Nevertheless, the magnitude of the volume loss observed in our study appears to exceed that reported for healthy aging, supporting the presence of early neurodegenerative changes during the RIS stage.

In a multicenter MS cohort study conducted by Meijboom et al., global and regional changes in brain volume were longitudinally assessed using MRI scans obtained at two different time points, similar to the approach used in our study [8]. The authors reported significant longitudinal volume reductions in total brain volume, brainstem volume, right and left cortical gray matter volumes, right and left cerebral white matter volumes, right and left cerebellar gray matter volumes, amygdala, basal ganglia, hippocampus, nucleus accumbens, thalamus, and ventral diencephalon. In our study, significant annualized volume loss was observed in total brain, total cerebrum, right cerebrum, left cerebrum, and total, right, and left thalamic volumes. The observation of significant volume loss in both total brain and thalamic volumes in the two studies suggests that these structures may be affected from the early stages of the demyelinating disease process. In contrast, the volume losses reported in other deep gray matter structures, cerebellar structures, and the brainstem in the MS cohort of Meijboom et al. was not observed in our RIS cohort. These differences may be attributable to the distinct stages of disease represented by MS and RIS, as well as differences in sample size and follow-up duration.

### 4.1. Total Brain Volume

Cross-sectional studies have demonstrated reduced total brain volume in patients with RIS at an early stage of the disease [12]. One study including patients with clinically isolated syndrome, RIS, and healthy controls found significant volume loss in both the RIS and clinically isolated syndrome groups compared with healthy controls, whereas no difference in the degree of atrophy was observed between the RIS and clinically isolated syndrome groups [7]. Similarly, another study comparing patients with RIS, MS, and healthy controls demonstrated that total brain volume was lower in patients with RIS than in healthy controls and was comparable to that observed in patients with MS [13]. These findings suggest that global brain volume loss may develop in patients with RIS even before the onset of clinical symptoms.

Unlike previous cross-sectional studies, our study longitudinally evaluated MRI scans obtained at two different time points in the same patients with RIS and calculated annualized percentage volume changes. Our results demonstrate that significant total brain volume loss occurs over time in patients with RIS. This finding suggests that the lower total brain volumes reported in cross-sectional studies may not solely reflect interindividual differences but may also represent measurable longitudinal volume loss within individuals. Thus, our study complements the existing cross-sectional literature by providing longitudinal evidence of the temporal progression of global brain atrophy during the RIS stage.

### 4.2. Cerebral White Matter and Cortical Gray Matter Volumes

Findings regarding cerebral white matter and cortical gray matter volumes in patients with RIS have been inconsistent. Some cross-sectional volumetric studies have reported reductions, particularly in cortical gray matter volume [5,7,12,13], whereas other studies have not identified significant differences in total white matter or cortical volumes [6,14]. These discrepancies may be related to differences in imaging protocols, segmentation methods, sample sizes, follow-up durations, and clinical characteristics of the study cohorts.

### 4.3. Subcortical Gray Matter

In our study, no significant volume changes were observed in total subcortical gray matter or in other subcortical structures, whereas a significant volume loss was observed exclusively in the bilateral thalami. This finding suggests that subcortical gray matter loss during the RIS stage may not affect all deep gray matter structures uniformly but may emerge earlier in specific anatomical regions, such as the thalamus.

The thalamus has also been reported to be among the earliest affected subcortical structures in patients with RIS. Cross-sectional volumetric studies have demonstrated significant reductions in thalamic volume, whereas findings concerning other subcortical gray matter structures have been more heterogeneous [5,14]. These findings suggest that the thalamus may be one of the earliest brain regions to exhibit structural changes during the RIS stage.

Longitudinal volumetric studies in patients with MS have likewise demonstrated that the thalamus is among the structures showing atrophy from the early stages of the disease, with this change potentially being more pronounced than that observed in many other subcortical structures. Similarly, in our study, significant volume loss was observed only in the thalami, suggesting that the thalamus may be particularly susceptible to involvement during the early stages of the demyelinating disease process. Given its extensive anatomical connections with cortical and subcortical structures, thalamic volume loss may represent a sensitive imaging marker of early structural changes within the central nervous system [8,15,16].

The thalamus is considered one of the major integration centers of the central nervous system because of its extensive anatomical connections with the cerebral cortex and subcortical structures [17]. In MS, thalamic atrophy is thought not to result solely from local thalamic pathology but rather from the combined effects of widespread axonal injury, Wallerian and transneuronal degeneration, and gray matter pathology [18]. The significant bilateral thalamic volume loss observed in our cohort of patients with RIS who had not yet developed a clinical demyelinating attack suggests that similar neurodegenerative processes may already be initiated during the preclinical stage of the disease. However, long-term prospective studies incorporating advanced imaging techniques are needed to better elucidate the underlying mechanisms of thalamic volume loss.

### 4.4. Infratentorial Structures

No significant volume loss was detected in the brainstem or cerebellum in our study. The number of studies evaluating infratentorial structures volumetrically in patients with RIS is limited. Most available studies have focused on supratentorial brain regions, while volumetric changes in the brainstem and cerebellum have not been extensively investigated [6,7,14].

Cerebellar atrophy in patients with MS has been reported to emerge predominantly during later stages of the disease and to be associated with impairments in motor coordination, balance, and cognitive function [19]. The absence of significant volume loss in infratentorial structures in our study may indicate that these regions remain relatively preserved during the RIS stage. Nevertheless, larger longitudinal studies with longer follow-up periods are required to characterize the temporal trajectory of volumetric changes in infratentorial structures.

In our study, no significant associations were observed between annualized brain volume changes and total T2 lesion count, regional T2 lesion counts, or total T2 lesion volume. Similarly, subgroup analyses based on MRI progression, the presence of contrast-enhancing lesions, and the presence of spinal cord lesions did not reveal significant differences in annualized volume changes. The literature regarding the relationship between conventional MRI lesion burden and longitudinal brain volume changes in RIS remains limited. In MS cohorts, the relationship between lesion burden and brain atrophy appears to be complex, and focal lesions detected on conventional MRI may not fully account for changes in brain volume [20,21]. Our findings may likewise be interpreted within this framework. Nevertheless, given the limited sample size, these results should be interpreted cautiously and warrant further evaluation in larger longitudinal cohorts.

The volBrain platform has demonstrated good test–retest reproducibility in previous validation studies. However, the pipeline used in this study was not specifically designed as a longitudinal processing stream; rather, the same volumetric analysis pipeline was applied separately to MRI scans obtained at the two time points. Therefore, potential measurement variability should be considered, particularly when interpreting small longitudinal volume changes [22,23].

## 5. Limitations

This study has several limitations. First, the relatively small sample size limits the generalizability of the findings. In addition, the proportion of female patients in our cohort was higher than that reported in previous RIS cohorts. This may reflect the demographic characteristics of our single-center sample and may limit the generalizability of our findings to other patient populations. Second, the retrospective design of the study and the absence of a healthy control group make it difficult to determine whether the observed volume changes are specifically attributable to RIS-related processes. Furthermore, measurement variability associated with the automated segmentation methods used for volumetric analysis should be considered, particularly when interpreting small volumetric changes. Finally, because clinical conversion data were unavailable, the relationship between the observed volumetric changes and subsequent development of MS could not be evaluated.

A further limitation is the potential measurement variability associated with longitudinal volumetric assessment, particularly for smaller brain structures. Although the observed annualized volume changes in the thalamus may represent genuine longitudinal structural changes, the relatively wide distribution of these changes indicates that measurement variability should also be considered, particularly when relatively short MRI intervals are annualized. Therefore, the observed changes in smaller structures should be interpreted with appropriate caution. In addition, the volBrain pipeline used in this study was not specifically designed as a longitudinal processing stream; rather, the same volumetric analysis pipeline was applied separately to MRI scans obtained at the two time points.

Future studies should include larger, multicenter cohorts with longitudinal follow-up. Comparative analyses across different automated segmentation software platforms should also be performed to account for potential platform-related effects. Diffusion-based measures and functional connectivity analyses capable of detecting tissue-level alterations should be integrated with volumetric data. Furthermore, multimodal approaches incorporating genetic, immunological, and fluid biomarkers may provide additional mechanistic insights into the pathophysiology of RIS.

## 6. Conclusions

This longitudinal volumetric study demonstrated significant annualized volume loss in total brain, cerebrum, and thalamic volumes in patients with RIS. No significant associations were observed between annualized volume changes and follow-up duration, lesion burden, or radiological subgroup characteristics. These findings contribute to the limited literature on longitudinal volumetric changes in RIS and expand current knowledge regarding structural brain alterations during the preclinical stage of the disease. Larger longitudinal studies incorporating appropriate control groups are warranted to further characterize the temporal trajectory of volumetric changes in RIS.

## Figures and Tables

**Table 1:** Demographic and Clinical Characteristics of Patients with RIS (*n* = 28).

Variable	*n* (%)/Median (IQR)
Sex	
Female	25 (89.3)
Male	3 (10.7)
DMT use	
Yes	0 (0.0)
No	28 (100)
Age at *t*_0_ (years)	41.00 (33.25–45.75)
Interval between *t*_0_ and *t*_1_ (months)	15.50 (9.50–24.75)
SDMT score (*n* = 22)	43.50 (30.25–51.25)
Timed 25-Foot Walk test (s) (*n* = 24)	11.43 (8.78–13.65)
Nine-Hole Peg Test, right hand (s) (*n* = 24)	20.81 (17.94–23.75)
Nine-Hole Peg Test, left hand (s) (*n* = 24)	20.65 (18.74–21.27)

Values are presented as *n* (%) or median (IQR). IQR, interquartile range (25th–75th percentile); DMT, disease-modifying therapy; *t*_0_, first time point; *t*_1_, second time point; SDMT, Symbol Digit Modalities Test.

**Table 2:** Longitudinal Changes in Global and Regional Brain Volumes.

	t_0_ Volume, cm^3^	t_1_ Volume, cm^3^	Annualized % Change	*p* Value	FDR-Adjusted *p* Value
Total brain volume	1189.8 (1114.3–1302.4)	1170.3 (1100.6–1287.3)	−1.1 (−1.7–−0.2)	0.003	0.043
Total cerebrum volume	1059.8 (984.9–1157.7)	1038.5 (971.4–1143.0)	−1.1 (−1.7–0.0)	0.001	0.033
Right cerebrum volume	534.5 (490.8–579.8)	520.5 (486.4–573.2)	−1.3 (−1.9–−0.3)	0.001	0.033
Left cerebrum volume	525.9 (497.5–576.5)	518.0 (485.8–570.3)	−1.1 (−1.7–0.0)	0.004	0.044
Total thalamic volume	12.2 (11.6–13.6)	11.7 (10.7–13.5)	−1.9 (−5.3–−0.3)	0.001	0.033
Right thalamic volume	6.1 (5.5–6.8)	5.8 (5.3–6.8)	−2.2 (−4.4–0.3)	0.005	0.047
Left thalamic volume	6.2 (5.8–6.7)	5.8 (5.3–6.7)	−2.6 (−8.1–−0.3)	0.003	0.043

Values are presented as median (IQR). The Wilcoxon signed-rank test was used to compare measurements between *t*_0_ and *t*_1_. IQR, interquartile range (25th–75th percentile); *t*_0_, first time point; *t*_1_, second time point; FDR, Benjamini–Hochberg false discovery rate correction.

**Table 3:** Correlations Between Age, the *t*_0_–*t*_1_ Interval, and Longitudinal Brain Volume Changes.

	Statistic	Age	*t*_0_–*t*_1_ Interval
Annualized Total Brain Volume Change (%)	r	−0.119	0.249
	*p*	0.545	0.201
	FDR-adjusted *p*	0.734	0.526
Annualized Total Cerebrum Volume Change (%)	r	−0.147	0.195
	*p*	0.455	0.319
	FDR-adjusted *p*	0.697	0.656
Annualized Right Cerebrum Volume Change (%)	r	−0.136	0.267
	*p*	0.491	0.17
	FDR-adjusted *p*	0.712	0.498
Annualized Left Cerebrum Volume Change (%)	r	−0.190	0.152
	*p*	0.334	0.439
	FDR-adjusted *p*	0.659	0.694
Annualized Total Thalamus Volume Change (%)	r	−0.250	0.026
	*p*	0.199	0.895
	FDR-adjusted *p*	0.526	0.987
Annualized Right Thalamus Volume Change (%)	r	−0.349	0.002
	*p*	0.068	0.99
	FDR-adjusted *p*	0.323	0.997
Annualized Left Thalamus Volume Change (%)	r	−0.150	0.115
	*p*	0.447	0.561
	FDR-adjusted *p*	0.697	0.749

*r*, Spearman correlation coefficient; FDR, Benjamini–Hochberg false discovery rate correction.

**Table 4:** MRI Lesion Characteristics of the RIS Cohort (*n* = 28).

MRI Characteristic	Value
Total lesion count, median (IQR)	21.5 (12.0–31.2)
Juxtacortical lesion count, median (IQR)	9.0 (4.0–17.5)
Periventricular lesion count, median (IQR)	9.5 (4.8–12.8)
Infratentorial lesion count, median (IQR)	0.0 (0.0–1.0)
Spinal cord lesion, *n* (%)	9 (32.1)
Total lesion volume (cm^3^), median (IQR)	4.3 (2.2–6.5)
MRI progression, *n* (%)	11 (39.3)
Contrast-enhancing lesion at baseline (*t*_0_), *n* (%)	5 (17.9)
Contrast-enhancing lesion at follow-up (*t*_1_), *n* (%)	7 (25.0)

IQR, interquartile range.

**Table 5:** Correlations Between MRI Lesion Characteristics and Annualized Brain Volume Changes.

		Total Lesion Count	Juxtacortical Lesion Count	Periventricular Lesion Count	Infratentorial Lesion Count	Total Lesion Volume (cm^3^)
Total Brain Volume Change (%)	r	0.071	−0.040	0.145	0.071	0.01
	*p*	0.72	0.839	0.461	0.72	0.958
	FDR-adjusted *p*	0.965	0.965	0.965	0.965	0.965
Total Cerebrum Volume Change (%)	r	0.096	−0.047	0.222	0.058	0.009
	*p*	0.628	0.813	0.257	0.769	0.965
	FDR-adjusted *p*	0.965	0.965	0.965	0.965	0.965
Right Cerebrum Volume Change (%)	r	0.05	−0.049	0.138	0.093	−0.017
	*p*	0.802	0.806	0.485	0.639	0.931
	FDR-adjusted *p*	0.965	0.965	0.965	0.965	0.965
Left Cerebrum Volume Change (%)	r	0.097	−0.096	0.265	0.033	−0.016
	*p*	0.624	0.627	0.173	0.868	0.935
	FDR-adjusted *p*	0.965	0.965	0.965	0.965	0.965
Total Thalamus Volume Change (%)	r	−0.021	−0.098	0.02	0.119	0.029
	*p*	0.915	0.618	0.919	0.547	0.882
	FDR-adjusted *p*	0.965	0.965	0.965	0.965	0.965
Right Thalamus Volume Change (%)	r	0.044	−0.090	0.053	0.038	−0.085
	*p*	0.825	0.648	0.787	0.849	0.666
	FDR-adjusted *p*	0.965	0.965	0.965	0.965	0.965
Left Thalamus Volume Change (%)	r	−0.073	−0.131	−0.058	0.316	0.05
	*p*	0.712	0.507	0.769	0.102	0.802
	FDR-adjusted *p*	0.965	0.965	0.965	0.965	0.965

r, Spearman correlation coefficient.

## Data Availability

The data that support the findings of this study are available from the corresponding author upon reasonable request.

## Supplementary Materials

The following supporting information can be downloaded at: https://www.mdpi.com/article/10.3390/medicina62101948/s1, Supplementary Table S1. Complete Results of Longitudinal Brain Volume Comparisons with Unadjusted and FDR-Adjusted *p*-Values; Supplementary Table S2. Brain Volume Changes According to MRI Progression; Supplementary Table S3. Brain Volume Changes According to Baseline (t_0_) Contrast-Enhancing Lesion Status; Supplementary Table S4. Brain Volume Changes According to Follow-up (t_1_) Contrast-Enhancing Lesion Status; Supplementary Table S5. Brain Volume Changes According to Spinal Cord Lesion Status.

## References

[1] Okuda D.T., Mowry E.M., Beheshtian A., Waubant E., Baranzini S.E., Goodin D.S., Hauser S.L., Pelletier D. (2009). Incidental MRI anomalies suggestive of multiple sclerosis: The radiologically isolated syndrome. Neurology.

[2] Okuda D.T., Siva A., Kantarci O., Inglese M., Katz I., Tutuncu M., Keegan B.M., Donlon S., Hua L.H., Vidal-Jordana A. (2014). Radiologically isolated syndrome: 5-year risk for an initial clinical event. PLoS ONE.

[3] Hosseiny M., Newsome S.D., Yousem D.M. (2020). Radiologically Isolated Syndrome: A Review for Neuroradiologists. AJNR Am. J. Neuroradiol..

[4] Lebrun-Frénay C., Rollot F., Mondot L., Zephir H., Louapre C., Le Page E., Durand-Dubief F., Labauge P., Bensa C., Thouvenot E. (2021). Risk Factors and Time to Clinical Symptoms of Multiple Sclerosis Among Patients with Radiologically Isolated Syndrome. JAMA Netw. Open.

[5] Labiano-Fontcuberta A., Mato-Abad V., Álvarez-Linera J., Hernández-Tamames J.A., Martínez-Ginés M.L., Aladro Y., Ayuso L., Domingo-Santos Á., Benito-León J. (2016). Gray Matter Involvement in Radiologically Isolated Syndrome. Medicine.

[6] George I.C., El Mendili M.M., Inglese M., Azevedo C.J., Kantarci O., Lebrun C., Siva A., Okuda D.T., Pelletier D. (2021). Cerebellar volume loss in radiologically isolated syndrome. Mult. Scler..

[7] Rojas J.I., Patrucco L., Míguez J., Besada C., Cristiano E. (2015). Brain atrophy in radiologically isolated syndromes. J. Neuroimaging.

[8] Meijboom R., York E.N., Kampaite A., Harris M.A., White N., Valdés Hernández M.D.C., Thrippleton M.J., MacDougall N.J.J., Connick P., Hunt D.P.J. (2023). Patterns of brain atrophy in recently-diagnosed relapsing-remitting multiple sclerosis. PLoS ONE.

[9] Reinhardt C., Angstwurm K., Freudenstein D., Lee D.H., Wendl C., Linker R.A. (2024). Real-world analysis of brain atrophy in multiple sclerosis patients with an artificial intelligence based software tool. Neurol. Res. Pract..

[10] Scahill R.I., Frost C., Jenkins R., Whitwell J.L., Rossor M.N., Fox N.C. (2003). A longitudinal study of brain volume changes in normal aging using serial registered magnetic resonance imaging. Arch. Neurol..

[11] Fotenos A.F., Snyder A.Z., Girton L.E., Morris J.C., Buckner R.L. (2005). Normative estimates of cross-sectional and longitudinal brain volume decline in aging and AD. Neurology.

[12] Labiano-Fontcuberta A., Mato-Abad V., Álvarez-Linera J., Hernández-Tamames J.A., Martínez-Ginés M.L., Aladro Y., Ayuso L., Domingo-Santos Á., Benito-León J. (2016). Normal-appearing brain tissue analysis in radiologically isolated syndrome using 3 T MRI. Medicine.

[13] Amato M.P., Hakiki B., Goretti B., Rossi F., Stromillo M.L., Giorgio A., Roscio M., Ghezzi A., Guidi L., Bartolozzi M.L. (2012). Association of MRI metrics and cognitive impairment in radiologically isolated syndromes. Neurology.

[14] Azevedo C.J., Overton E., Khadka S., Buckley J., Liu S., Sampat M., Kantarci O., Lebrun Frenay C., Siva A., Okuda D.T. (2015). Early CNS neurodegeneration in radiologically isolated syndrome. Neurol. Neuroimmunol. Neuroinflamm..

[15] Kılıç B., Tekin A., Bünül S.D., Efendi H., Çakır Ö., Kılıç K.C., Çolak T. (2025). Subcortical volume alterations indicate structural atrophy patterns over time in relapsing-remitting multiple sclerosis: A volBrain-based analysis. Ir. J. Med. Sci..

[16] Tekin A., Rende B., Efendi H., Bunul S.D., Çakır Ö., Çolak T., Balcı S. (2024). Volumetric and Asymmetric Index Analysis of Subcortical Structures in Multiple Sclerosis Patients: A Retrospective Study Using volBrain Software. Cureus.

[17] Hwang K., Bertolero M.A., Liu W.B., D’Esposito M. (2017). The Human Thalamus Is an Integrative Hub for Functional Brain Networks. J. Neurosci..

[18] Kipp M., Wagenknecht N., Beyer C., Samer S., Wuerfel J., Nikoubashman O. (2015). Thalamus pathology in multiple sclerosis: From biology to clinical application. Cell. Mol. Life Sci..

[19] Ozturk O., Ozturk S., Gul S., Bunul S.D., Efendi H., Cam I. (2021). The relationship between cerebellar volume, clinical disability and cognitive changes in multiple sclerosis patients. Neurol. Asia.

[20] Chard D.T., Brex P.A., Ciccarelli O., Griffin C.M., Parker G.J., Dalton C., Altmann D.R., Thompson A.J., Miller D.H. (2003). The longitudinal relation between brain lesion load and atrophy in multiple sclerosis: A 14 year follow up study. J. Neurol. Neurosurg. Psychiatry.

[21] Ge Y., Grossman R.I., Udupa J.K., Wei L., Mannon L.J., Polansky M., Kolson D.L. (2000). Brain atrophy in relapsing-remitting multiple sclerosis and secondary progressive multiple sclerosis: Longitudinal quantitative analysis. Radiology.

[22] Manjón J.V., Coupé P. (2016). volBrain: An Online MRI Brain Volumetry System. Front. Neuroinform..

[23] Coupé P., Mansencal B., Clément M., Giraud R., de Senneville B.D., Ta V.T., Lepetit V., Manjon J.V. (2020). AssemblyNet: A large ensemble of CNNs for 3D whole brain MRI segmentation. NeuroImage.

