# Supplementary material for: Temporal Change: Evaluation of Brain Atrophy in Patients with Radiologically Isolated Syndrome Using volBrain
Source: Medicina (Kaunas). 2026 Oct 9;62(10):1948. doi: 10.3390/medicina62101948 (PMC13706695; doi:10.3390/medicina62101948)
Supplement: Supplementary file 1 [file medicina-62-01948-s001.zip › medicina-4588984-supplementary.pdf]

Supplementary Table S1. Complete Results of Longitudinal Brain Volume Comparisons with Unadjusted and FDR-Adjusted p-Values

**Region**	**t0**	**t1**	**Annualized Change (%)**	**p**	**FDR-adjusted p**
Total White Matter Volume	535.4 (489.4-563.8)	519.5 (468.3-550.3)	-1.3 ((-5.1)-1.1)	**0.042**	0.230
Total Gray Matter Volume	667.1 (625.7-723.3)	663.1 (631.0-727.0)	-0.2 ((-2.9)-2.0)	0.531	0.990
Total Subcortical Gray Matter Volume	41.2 (37.1-44.2)	40.4 (37.0-43.8)	-0.3 ((-2.4)-3.8)	0.516	0.990
Total Cortical Gray Matter Volume	528.9 (491.9-571.8)	521.4 (494.5-575.1)	-1.0 ((-2.9)-2.3)	0.561	0.990
Total Cerebellar Gray Matter Volume	102.7 (96.3-109.2)	103.1 (93.9-111.0)	-1.1 ((-3.0)-3.0)	0.459	0.990
Total Cerebrospinal Fluid Volume	85.8 (67.9-109.1)	96.5 (70.1-119.7)	5.8 ((-4.6)-14.8)	**0.023**	0.188
Total Brain Volume	1189.8 (1114.3-1302.4)	1170.3 (1100.6-1287.3)	-1.1 ((-1.7)-(-0.2))	**0.003**	**0.043**
Total Cerebrum Volume	1059.8 (984.9-1157.7)	1038.5 (971.4-1143.0)	-1.1 ((-1.7)-0.0)	**0.001**	**0.033**
Total Cerebrum White Matter Volume	502.0 (461.3-533.3)	487.0 (437.2-521.1)	-1.1 ((-5.3)-0.9)	**0.030**	0.194
Total Cerebrum Gray Matter Volume	564.6 (530.5-614.0)	560.1 (533.6-618.5)	-0.7 ((-2.9)-2.1)	0.561	0.990
Right Cerebrum Volume	534.5 (490.8-579.8)	520.5 (486.4-573.2)	-1.3 ((-1.9)-(-0.3))	**0.001**	**0.033**
Right Cerebrum White Matter Volume	252.2 (229.9-269.1)	248.3 (221.3-260.2)	-1.3 ((-5.7)-0.7)	**0.022**	0.188
Right Cerebrum Gray Matter Volume	282.1 (266.8-307.8)	280.2 (265.3-310.5)	-0.7 ((-3.0)-2.1)	0.593	0.990
Left Cerebrum Volume	525.9 (497.5-576.5)	518.0 (485.8-570.3)	-1.1 ((-1.7)-0.0)	**0.004**	**0.044**
Left Cerebrum White Matter Volume	251.9 (227.2-264.0)	242.4 (215.5-260.9)	-1.3 ((-4.8)-1.2)	0.052	0.265
Left Cerebrum Gray Matter Volume	286.0 (263.7-305.7)	280.9 (267.9-308.0)	-0.5 (-2.7-2.1)	0.641	0.990
Total Cerebellum Volume	123.8 (119.6-135.0)	125.9 (116.0-135.9)	-0.8 ((-2.1)-1.4)	0.260	0.935
Total Cerebellum White Matter Volume	30.6 (26.5-34.2)	30.3 (28.1-33.4)	-1.1 ((-5.7)-3.0)	0.593	0.990
Total Cerebellum Gray Matter Volume	102.7 (96.3-109.2)	103.1 (93.9-111.0)	-1.1 ((-3.0)-3.0)	0.459	0.990
Right Cerebellum Volume	61.7 (59.2-67.7)	63.2 (57.9-68.0)	-0.7 ((-2.5)-1.4)	0.290	0.948
Right Cerebellum White Matter Volume	15.3 (13.3-17.4)	15.3 (14.2-16.6)	-0.4 ((-6.4)-2.8)	0.674	0.990
Right Cerebellum Gray Matter Volume	46.9 (43.8-50.6)	47.6 (42.7-51.8)	-1.1 ((-4.2)-2.3)	0.356	0.990
Left Cerebellum Volume	62.1 (59.1-66.8)	62.4 (57.7-68.0)	-0.7 ((-2.1)-1.3)	0.322	0.990
Left Cerebellum White Matter Volume	15.0 (13.2-16.8)	15.0 (13.9-16.6)	-0.6 ((-5.7)-3.6)	0.674	0.990
Left Cerebellum Gray Matter Volume	48.2 (44.6-50.5)	47.6 (43.0-51.0)	-0.7 ((-2.8)-2.8)	0.473	0.990
Total Vermis Volume	8.4 (8.1-8.8)	8.3 (7.8-9.0)	0.2 ((-2.6)-3.1)	0.982	1.000
Total Brainstem Volume	19.7 (18.1-22.6)	19.7 (17.4-22.2)	-0.9 ((-1.5)-(-0.3))	**0.030**	0.194
Total Accumbens Volume	0.6 (0.5-0.7)	0.6 (0.6-0.7)	-0.6 ((-5.5)-6.5)	0.619	0.990
Right Accumbens Volume	0.3 (0.3-0.3)	0.3 (0.3-0.3)	0.0 ((-5.3)-8.5)	0.733	1.000
Left Accumbens Volume	0.3 (0.3-0.4)	0.3 (0.3-0.3)	-3.0 ((-7.3)-1.7)	0.083	0.398

**Region**	**t0**	**t1**	**Annualized Change (%)**	**p**	**FDR-adjusted p**
Total Amygdala Volume	1.9 (1.8-2.1)	1.9 (1.8-2.2)	1.1 ((-2.3)-5.8)	0.387	0.990
Right Amygdala Volume	0.9 (0.9-1.0)	1.0 (0.9-1.1)	1.8 ((-2.2)-7.5)	0.289	0.948
Left Amygdala Volume	1.0 (0.9-1.0)	1.0 (0.9-1.1)	-0.4 ((-4.0)-8.3)	1.000	1.000
Total Basal Forebrain Volume	0.6 (0.5-0.7)	0.6 (0.5-0.7)	0.7 ((-7.3)-11.7)	0.838	1.000
Right Basal Forebrain Volume	0.3 (0.3-0.4)	0.3 (0.3-0.4)	3.2 ((-10.7)-12.7)	0.847	1.000
Left Basal Forebrain Volume	0.3 (0.2-0.3)	0.3 (0.2-0.4)	-1.1 ((-9.3)-9.5)	0.608	0.990
Total Caudate Volume	6.7 (6.0-7.3)	6.8 (6.1-7.4)	-0.1 ((-2.3)-3.6)	0.964	1.000
Right Caudate Volume	3.4 (3.1-3.7)	3.5 (3.1-3.7)	-0.1 ((-2.2)-3.2)	0.809	1.000
Left Caudate Volume	3.3 (3.0-3.7)	3.3 (3.0-3.7)	0.2 ((-2.5)-4.7)	0.982	1.000
Total Hippocampus Volume	7.7 (7.0-8.5)	7.6 (7.1-8.5)	0.1 ((-2.0)-4.1)	0.699	1.000
Right Hippocampus Volume	3.9 (3.6-4.3)	3.8 (3.6-4.3)	-0.1 ((-2.8)-4.6)	0.857	1.000
Left Hippocampus Volume	3.7 (3.4-4.1)	3.7 (3.4-4.2)	0.0 ((-2.4)-3.5)	0.980	1.000
Total Pallidum Volume	2.8 (2.6-3.1)	2.9 (2.7-3.1)	-0.2 ((-3.2)-2.6)	0.923	1.000
Right Pallidum Volume	1.4 (1.3-1.6)	1.5 (1.2-1.6)	0.0 ((-5.5)-2.8)	0.438	0.990
Left Pallidum Volume	1.4 (1.3-1.6)	1.4 (1.3-1.6)	0.8 ((-2.0)-3.4)	0.155	0.589
Total Putamen Volume	8.2 (7.2-8.7)	8.3 (7.1-8.9)	-0.4 ((-3.4)-2.3)	0.632	0.990
Right Putamen Volume	4.2 (3.7-4.4)	4.1 (3.6-4.4)	-0.9 ((-3.7)-0.9)	0.146	0.584
Left Putamen Volume	4.2 (3.6-4.4)	4.2 (3.5-4.4)	0.8 ((-3.8)-2.3)	1.000	1.000
Total Thalamus Volume	12.2 (11.6-13.6)	11.7 (10.7-13.5)	-1.9 ((-5.3)-(-0.3))	**0.001**	**0.033**
Right Thalamus Volume	6.1 (5.5-6.8)	5.8 (5.3-6.8)	-2.2 ((-4.4)-0.3)	**0.005**	**0.047**
Left Thalamus Volume	6.2 (5.8-6.7)	5.8 (5.3-6.7)	-2.6 ((-8.1)-(-0.3))	**0.003**	**0.043**
Total Ventral Diencephalon Volume	10.0 (9.0-10.9)	10.1 (9.3-10.8)	-0.4 ((-1.6)-1.1)	0.639	0.990
Right Ventral Diencephalon Volume	4.9 (4.4-5.4)	5.0 (4.6-5.3)	0.3 ((-1.9)-2.0)	0.764	1.000
Left Ventral Diencephalon Volume	5.1 (4.7-5.4)	5.1 (4.7-5.4)	-0.1 ((-1.8)-0.9)	0.624	0.990
Total Cortical Frontal Lobe Volume	189.4 (169.7-203.1)	187.4 (169.9-200.3)	-1.4 ((-2.8)-1.1)	0.094	0.424
Right Cortical Frontal Lobe Volume	96.3 (84.1-102.2)	94.4 (85.7-100.4)	-1.3 ((-2.7)-1.0)	**0.039**	0.230
Left Cortical Frontal Lobe Volume	92.8 (85.6-100.5)	93.0 (83.9-100.5)	-1.3 ((-3.2)-1.2)	0.119	0.503
Total Cortical Temporal Lobe Volume	108.9 (101.2-117.2)	107.3 (100.8-117.8)	-0.6 ((-2.5)-2.3)	0.577	0.990
Right Cortical Temporal Lobe Volume	55.4 (50.4-59.4)	54.6 (50.5-59.5)	-0.9 ((-3.1)-2.0)	0.345	0.990
Left Cortical Temporal Lobe Volume	54.0 (50.4-57.7)	53.5 (50.8-58.3)	-0.5 ((-2.6)-3.5)	0.927	1.000
Total Cortical Parietal Lobe Volume	103.5 (95.5-109.0)	100.9 (93.5-112.0)	-0.6 ((-2.3)-2.9)	0.829	1.000
Right Cortical Parietal Lobe Volume	51.8 (47.0-54.5)	50.8 (47.1-56.6)	-0.9 ((-2.6)-2.7)	0.776	1.000
Left Cortical Parietal Lobe Volume	51.6 (48.0-54.5)	50.4 (46.8-55.9)	-1.1 ((-2.4)-3.3)	0.657	0.990
Total Cortical Occipital Lobe Volume	65.5 (61.2-71.9)	67.0 (59.1-73.4)	0.4 ((-4.6)-5.7)	0.909	1.000
Right Cortical Occipital Lobe Volume	32.7 (30.3-36.2)	32.7 (28.7-37.1)	0.1 ((-5.0)-6.5)	0.829	1.000

Region	t0	t1	Annualized Change (%)	p	FDR-adjusted p
Left Cortical Occipital Lobe Volume	33.2 (30.8-35.4)	34.0 (30.4-36.8)	0.2 ((-3.8)-5.8)	0.936	1.000
Total Limbic Cortex Volume	38.4 (34.7-41.4)	39.0 (34.2-42.9)	0.0 ((-4.0)-7.5)	0.473	0.990
Right Limbic Cortex Volume	18.5 (16.5-20.0)	18.7 (16.4-20.6)	0.2 ((-4.1)-6.7)	0.624	0.990
Left Limbic Cortex Volume	19.7 (17.5-22.2)	19.9 (17.0-22.9)	0.1 ((-3.7)-5.1)	0.459	0.990
Total Insular Cortex Volume	28.2 (27.4-31.5)	28.4 (26.6-32.7)	-0.5 ((-1.8)-2.7)	0.811	1.000
Right Insular Cortex Volume	13.7 (12.9-15.5)	14.0 (12.7-16.0)	-0.7 ((-2.0)-3.0)	0.785	1.000
Left Insular Cortex Volume	15.0 (13.7-16.2)	14.8 (13.8-16.8)	0.1 ((-2.1)-3.1)	0.991	1.000

Values are presented as median (IQR). The Wilcoxon signed-rank test was used to compare measurements between t_0 and t_1. IQR, interquartile range (25th–75th percentile); t_0, first time point; t_1, second time point; FDR, Benjamini–Hochberg false discovery rate correction.

Supplementary Table S2. Brain Volume Changes According to MRI Progression

	MRI progression present (n = 11)	MRI progression absent (n = 17)	p value	FDR-adjusted p value
Total Brain Volume (t_0)	1192.8 (1116.1–1301.9)	1186.9 (1111.4–1306.7)	0.962	0.962
Total Brain Volume (t_1)	1145.5 (1098.0–1289.0)	1172.8 (1097.8–1291.6)	0.742	0.993
Within-group p	**0.011**	0.118		
Within-group FDR-adjusted p	**0.034**	0.118		
Total Brain Volume Change (%)	−1.4 ((−2.4)–(−0.5))	−0.8 ((−1.6)–0.3)	0.145	0.387
Total Cerebrum Volume (t_0)	1065.7 (990.3–1149.5)	1053.8 (980.0–1161.3)	0.888	0.962
Total Cerebrum Volume (t_1)	1019.4 (972.3–1142.4)	1041.3 (965.3–1145.0)	0.851	0.993
Within-group p	**0.014**	0.047		
Within-group FDR-adjusted p	**0.034**	0.073		
Total Cerebrum Volume Change (%)	−1.4 ((−2.2)–(−0.8))	−0.7 ((−1.5)–0.2)	0.173	0.387
Right Cerebrum Volume (t_0)	538.5 (499.8–580.5)	529.1 (481.5–580.1)	0.742	0.962
Right Cerebrum Volume (t_1)	516.7 (486.4–575.0)	521.8 (482.1–572.8)	1	1
Within-group p	**0.014**	0.033		
Within-group FDR-adjusted p	**0.034**	0.058		
Right Cerebrum Volume Change (%)	−1.5 ((−2.4)–(−1.3))	−1.0 ((−1.6)–0.0)	0.121	0.387
Left Cerebrum Volume (t_0)	527.2 (495.2–569.0)	524.6 (499.5–580.2)	0.888	0.962
Left Cerebrum Volume (t_1)	502.6 (485.7–567.4)	519.5 (487.1–573.1)	0.672	0.993
Within-group p	**0.014**	0.108		
Within-group FDR-adjusted p	**0.034**	0.116		
Left Cerebrum Volume Change (%)	−1.4 ((−1.9)–(−0.3))	−0.9 ((−1.3)–0.3)	0.221	0.387
Total Thalamus Volume (t_0)	11.9 (11.6–12.3)	12.6 (11.4–14.2)	0.151	0.529
Total Thalamus Volume (t_1)	11.6 (10.9–11.8)	12.3 (10.5–13.9)	0.24	0.754
Within-group p	**0.007**	0.058		

Within-group FDR-adjusted p	**0.034**	0.074		
Total Thalamus Volume Change (%)	−2.0 ((−5.7)–(−0.7))	−1.8 ((−4.7)–0.3)	0.397	0.556
Right Thalamus Volume (t_0)	6.0 (5.5–6.2)	6.3 (5.6–7.0)	0.23	0.537
Right Thalamus Volume (t_1)	5.7 (5.4–6.1)	6.0 (5.1–6.9)	0.323	0.754
Within-group p	**0.019**	0.08		
Within-group FDR-adjusted p	**0.038**	0.093		
Right Thalamus Volume Change (%)	−2.2 ((−3.7)–(−0.6))	−1.9 ((−5.1)–0.6)	0.778	0.778
Left Thalamus Volume (t_0)	5.9 (5.8–6.2)	6.5 (5.8–7.2)	0.082	0.529
Left Thalamus Volume (t_1)	5.5 (5.3–6.1)	6.3 (5.3–7.0)	0.18	0.754
Within-group p	**0.014**	0.058		
Within-group FDR-adjusted p	**0.034**	0.074		
Left Thalamus Volume Change (%)	−3.5 ((−8.5)–(−0.9))	−2.2 ((−7.4)–1.7)	0.541	0.631

Values are presented as median (IQR). The Mann–Whitney U test was used for between-group comparisons, and the Wilcoxon signed-rank test was used for within-group comparisons.

Supplementary Table S3. Brain Volume Changes According to Baseline (t_0) Contrast-Enhancing Lesion Status

	Present (n = 5)	Absent (n = 23)	p value	FDR-adjusted p value
Total Brain Volume (t_0)	1204.0 (946.9–1279.5)	1186.9 (1113.7–1304.1)	0.675	0.719
Total Brain Volume (t_1)	1171.2 (933.3–1253.2)	1169.3 (1108.2–1296.2)	0.509	0.594
Within-group p	0.178	**0.009**		
Within-group FDR-adjusted p	0.178	**0.031**		
Total Brain Volume Change (%)	−2.0 ((−4.3)–0.5)	−0.9 ((−1.6)–(−0.2))	0.168	0.436
Total Cerebrum Volume (t_0)	1080.5 (838.4–1131.3)	1053.8 (983.2–1162.1)	0.675	0.719
Total Cerebrum Volume (t_1)	1048.5 (827.5–1111.7)	1035.7 (972.3–1146.7)	0.509	0.594
Within-group p	0.178	**0.005**		
Within-group FDR-adjusted p	0.178	**0.031**		
Total Cerebrum Volume Change (%)	−1.6 ((−3.8)–0.4)	−1.1 ((−1.6)–0.0)	0.337	0.472
Right Cerebrum Volume (t_0)	542.8 (423.8–571.2)	534.1 (487.8–582.3)	0.719	0.719
Right Cerebrum Volume (t_1)	526.0 (416.3–559.4)	519.2 (486.5–573.7)	0.631	0.631
Within-group p	0.106	**0.003**		
Within-group FDR-adjusted p	0.134	**0.031**		
Right Cerebrum Volume Change (%)	−1.9 ((−4.2)–(−0.2))	−1.2 ((−1.5)–(−0.3))	0.187	0.436
Left Cerebrum Volume (t_0)	537.6 (414.6–560.1)	524.6 (503.6–582.2)	0.472	0.719
Left Cerebrum Volume (t_1)	522.5 (411.2–552.3)	516.5 (485.9–575.0)	0.401	0.594
Within-group p	0.178	**0.011**		
Within-group FDR-adjusted p	0.178	**0.031**		
Left Cerebrum Volume Change (%)	−1.5 ((−3.4)–1.1)	−1.0 ((−1.4)–0.0)	0.472	0.55
Total Thalamus Volume (t_0)	11.2 (10.1–13.2)	12.3 (11.7–13.8)	0.219	0.51

Total Thalamus Volume (t_1)	10.9 (9.2–12.7)	11.8 (10.9–13.5)	0.187	0.594
Within-group p	0.059	**0.009**		
Within-group FDR-adjusted p	0.083	**0.031**		
Total Thalamus Volume Change (%)	−4.2 ((−6.9)–(−2.1))	−1.1 ((−3.5)–(−0.2))	0.119	0.436
Right Thalamus Volume (t_0)	5.4 (4.9–6.6)	6.1 (5.7–6.8)	0.168	0.51
Right Thalamus Volume (t_1)	5.4 (4.6–6.4)	5.9 (5.4–6.8)	0.337	0.594
Within-group p	0.059	**0.026**		
Within-group FDR-adjusted p	0.083	0.052		
Right Thalamus Volume Change (%)	−2.2 ((−5.0)–(−1.7))	−2.2 ((−4.7)–0.5)	0.631	0.631
Left Thalamus Volume (t_0)	5.8 (5.2–6.6)	6.3 (5.8–6.8)	0.15	0.51
Left Thalamus Volume (t_1)	5.5 (4.6–6.3)	5.9 (5.3–6.7)	0.187	0.594
Within-group p	0.059	**0.016**		
Within-group FDR-adjusted p	0.083	**0.036**		
Left Thalamus Volume Change (%)	−6.0 ((−8.9)–(−2.5))	−1.8 ((−7.2)–1.3)	0.28	0.472

Values are presented as median (IQR). The Mann–Whitney U test was used for between-group comparisons, and the Wilcoxon signed-rank test was used for within-group comparisons.

Supplementary Table S4. Brain Volume Changes According to Follow-up (t_1) Contrast-Enhancing Lesion Status

	Present (n = 7)	Absent (n = 21)	p value	FDR-adjusted p value
Total Brain Volume (t_0)	1192.8 (1100.2–1302.6)	1186.9 (1114.9–1303.0)	1	1
Total Brain Volume (t_1)	1132.1 (1098.0–1296.2)	1172.8 (1097.8–1285.7)	0.791	0.915
Within-group p	0.076	**0.016**		
Within-group FDR-adjusted p	0.097	**0.04**		
Total Brain Volume Change (%)	−1.3 ((−1.5)–(−0.2))	−1.1 ((−1.8)–(−0.1))	0.915	0.915
Total Cerebrum Volume (t_0)	1065.7 (982.0–1167.9)	1053.8 (986.7–1155.0)	0.958	1
Total Cerebrum Volume (t_1)	1003.9 (972.3–1159.5)	1041.3 (965.3–1142.8)	0.915	0.915
Within-group p	0.108	**0.006**		
Within-group FDR-adjusted p	0.108	**0.04**		
Total Cerebrum Volume Change (%)	−1.4 ((−1.6)–0.0)	−1.1 ((−1.7)–(−0.1))	0.832	0.915
Right Cerebrum Volume (t_0)	538.5 (486.7–584.8)	534.1 (493.8–579.2)	0.874	1
Right Cerebrum Volume (t_1)	506.8 (486.4–577.9)	521.8 (483.3–572.8)	0.915	0.915
Within-group p	0.108	**0.004**		
Within-group FDR-adjusted p	0.108	**0.04**		
Right Cerebrum Volume Change (%)	−1.4 ((−1.7)–0.1)	−1.2 ((−1.9)–(−0.3))	0.874	0.915
Left Cerebrum Volume (t_0)	527.2 (495.2–583.1)	524.6 (499.5–574.7)	1	1
Left Cerebrum Volume (t_1)	497.1 (485.7–581.5)	519.5 (487.1–569.4)	0.832	0.915
Within-group p	0.076	**0.018**		
Within-group FDR-adjusted p	0.097	**0.04**		

Left Cerebrum Volume Change (%)	−1.4 ((−1.8)–0.0)	−1.0 ((−1.5)–0.2)	0.71	0.915
Total Thalamus Volume (t_0)	11.8 (11.6–12.2)	12.6 (11.4–13.9)	0.089	0.304
Total Thalamus Volume (t_1)	11.6 (10.7–11.8)	11.9 (10.8–13.8)	0.265	0.619
Within-group p	0.034	**0.014**		
Within-group FDR-adjusted p	0.06	**0.04**		
Total Thalamus Volume Change (%)	−1.1 ((−8.1)–(−0.7))	−2.3 ((−4.9)–(−0.2))	0.832	0.915
Right Thalamus Volume (t_0)	5.9 (5.5–6.1)	6.3 (5.6–6.9)	0.13	0.304
Right Thalamus Volume (t_1)	5.4 (5.3–5.9)	6.0 (5.3–6.8)	0.185	0.619
Within-group p	0.093	**0.02**		
Within-group FDR-adjusted p	0.108	**0.04**		
Right Thalamus Volume Change (%)	−2.2 ((−7.0)–0.0)	−2.2 ((−4.1)–0.4)	0.832	0.915
Left Thalamus Volume (t_0)	5.9 (5.7–6.2)	6.4 (5.8–7.1)	0.1	0.304
Left Thalamus Volume (t_1)	5.7 (5.2–6.1)	5.8 (5.3–6.9)	0.233	0.619
Within-group p	0.076	**0.014**		
Within-group FDR-adjusted p	0.097	**0.04**		
Left Thalamus Volume Change (%)	−1.8 ((−9.3)–(−0.9))	−2.9 ((−7.2)–0.5)	0.791	0.915

Values are presented as median (IQR). The Mann–Whitney U test was used for between-group comparisons, and the Wilcoxon signed-rank test was used for within-group comparisons.

Supplementary Table S5. Brain Volume Changes According to Spinal Cord Lesion Status

	Present (n = 9)	Absent (n = 19)	p value	FDR-adjusted p value
Total Brain Volume (t_0)	1257.2 (1161.9–1324.0)	1174.0 (1100.2–1289.4)	0.094	0.22
Total Brain Volume (t_1)	1217.3 (1119.4–1327.8)	1157.0 (1087.3–1275.5)	0.219	0.383
Within-group p	0.097	**0.012**		
Within-group FDR-adjusted p	0.105	**0.033**		
Total Brain Volume Change (%)	−1.5 ((−2.2)–0.1)	−0.8 ((−1.6)–(−0.2))	0.279	0.694
Total Cerebrum Volume (t_0)	1113.1 (1038.6–1180.2)	1042.2 (976.9–1147.6)	0.085	0.22
Total Cerebrum Volume (t_1)	1081.0 (998.9–1181.8)	1026.7 (959.5–1139.9)	0.168	0.383
Within-group p	0.076	**0.009**		
Within-group FDR-adjusted p	0.105	**0.033**		
Total Cerebrum Volume Change (%)	−1.4 ((−1.9)–0.0)	−1.1 ((−1.6)–0.0)	0.523	0.694
Right Cerebrum Volume (t_0)	561.9 (522.2–592.1)	522.3 (485.9–576.3)	0.069	0.22
Right Cerebrum Volume (t_1)	543.9 (502.0–592.4)	516.7 (477.6–571.8)	0.14	0.383
Within-group p	0.076	**0.007**		
Within-group FDR-adjusted p	0.105	**0.033**		
Right Cerebrum Volume Change (%)	−1.7 ((−2.2)–0.0)	−1.2 ((−1.5)–(−0.3))	0.325	0.694
Left Cerebrum Volume (t_0)	551.1 (516.4–588.1)	515.8 (495.2–571.3)	0.154	0.269
Left Cerebrum Volume (t_1)	537.1 (496.9–589.4)	513.5 (484.6–566.2)	0.219	0.383
Within-group p	0.097	**0.017**		

Within-group FDR-adjusted p	0.105	**0.033**		
Left Cerebrum Volume Change (%)	−1.4 ((−1.8)–0.1)	−1.0 ((−1.3)–0.0)	0.623	0.694
Total Thalamus Volume (t_0)	12.3 (10.6–13.9)	12.2 (11.7–13.3)	0.731	0.731
Total Thalamus Volume (t_1)	11.7 (10.6–13.9)	11.8 (10.9–13.4)	0.922	1
Within-group p	0.058	**0.007**		
Within-group FDR-adjusted p	0.102	**0.033**		
Total Thalamus Volume Change (%)	−1.0 ((−4.9)–(−0.2))	−2.3 ((−5.9)–(−0.3))	0.694	0.694
Right Thalamus Volume (t_0)	6.1 (5.2–6.8)	6.0 (5.6–6.8)	0.676	0.731
Right Thalamus Volume (t_1)	6.1 (5.4–6.8)	5.8 (5.2–6.8)	0.768	1
Within-group p	0.183	**0.016**		
Within-group FDR-adjusted p	0.183	**0.033**		
Right Thalamus Volume Change (%)	−2.2 ((−3.3)–0.4)	−2.7 ((−5.5)–0.4)	0.491	0.694
Left Thalamus Volume (t_0)	6.2 (5.4–7.2)	6.3 (5.8–6.6)	0.623	0.731
Left Thalamus Volume (t_1)	5.5 (5.2–7.1)	5.8 (5.3–6.6)	1	1
Within-group p	0.097	**0.015**		
Within-group FDR-adjusted p	0.105	**0.033**		
Left Thalamus Volume Change (%)	−1.3 ((−7.2)–0.8)	−3.4 ((−10.2)–(−0.4))	0.461	0.694

Values are presented as median (IQR). The Mann–Whitney U test was used for between-group comparisons, and the Wilcoxon signed-rank test was used for within-group comparisons.
